# Pregnancy-related maternal and fetal outcomes following renal transplantation: a decade of experience at a tertiary care center in Saudi Arabia

**DOI:** 10.1186/s12884-025-08385-3

**Published:** 2025-11-18

**Authors:** Judy Hejazi, Ahmad Aljumaa, Reem Elmokattaf, Jihad Aljumaa, Waleed A. AL-Amoudi, Sarah A. AL-Amoudi, Roaa Aljumaa, Sameer Desai, M. Mohanad Al Hennawi, Maisoon Almugbel

**Affiliations:** 1 College of Medicine, Alfaisal University, Riyadh, Saudi Arabia; 2 Stepping Hill Hospital, Stockport, UK; 3 College of Medicine, Dar Al-Uloom University, Riyadh, Saudi Arabia; 4 Department of Biostatistics, Epidemiology, and Scientific Computing, King Faisal Specialist Hospital & Research Center, Riyadh, Saudi Arabia; 5 Department of Obstetrics & Gynecology, King Faisal Specialist Hospital & Research Center, Riyadh, Saudi Arabia

**Keywords:** Renal transplant, Kidney transplant, Fetal outcomes, Maternal outcomes, EGFR, Pregnancy, Graft rejection

## Abstract

**Background:**

Advancements in immunosuppressive therapy and transplant care have made pregnancy increasingly feasible for women with renal transplants. We aim to evaluate maternal and neonatal outcomes of pregnancies in kidney recipients followed up at a tertiary care center in Saudi Arabia.

**Methods:**

A retrospective analysis was performed of women with kidney transplants who became pregnant between 2012 and 2021. Maternal demographics, transplant characteristics, immunosuppressive regimens, renal function parameters, pregnancy complications, and neonatal outcomes were assessed.

**Results:**

A total of 37 pregnancies in 35 kidney transplant recipients were evaluated with 2 resulting in miscarriage. The majority of transplants originated from living (88%) and related (87%) donors. Pre-pregnancy labs showed anemia in 26%, elevated creatinine (> 90 µmol/L) in 23%, eGFR < 60 mL/min/1.73 m² in 17%, and proteinuria in 26%. All patients had normal platelets and liver function tests, with a mean tacrolimus level of 7.4 ± 2.6 ng/mL. At the time of labor, the proportion of patients with anemia increased to 66%, while abnormal white blood cell counts and thrombocytopenia were observed in 15% and 21% of patients, respectively. Elevated creatinine (> 90 µmol/L) was present in 43%, eGFR < 60 mL/min/1.73 m² in 40%, and proteinuria in 37%. Liver function tests remained normal in all patients. While most patients demonstrated substantial recovery in renal function, a minority (21%) exhibited persistent impairment up to two years following delivery. Cesarean sections were performed in 74% of cases, predominantly due to fetal distress. Neonatal outcomes showed that 63% of infants were born with normal birth weight, and the mean gestational age at delivery was 35 weeks, with 57% of pregnancies reaching term. Of the 35 born neonates, admission to the neonatal intensive care unit was required in 9 (26%) with congenital anomalies observed in 1 (3%).

**Conclusion:**

Pregnancy after kidney transplantation carries inherent risks but, with vigilant monitoring and multidisciplinary care, can achieve favorable outcomes. Although transient declines in renal function may occur, most patients in this study maintained stable long-term graft function. Maternal and neonatal complication rates remain higher than in the general population, highlighting the importance of individualized care and comprehensive preconception counseling.

## Background

In cases of end-stage renal disease (ESRD), renal transplantation is the preferred treatment, as it not only improves survival but also significantly enhances quality of life. Among all solid organ transplants, the kidney remains the most frequently transplanted organ [1]. In women with end-stage renal disease (ESRD), disruption of the hypothalamic–pituitary–gonadal axis leads to impaired fertility. Consequently, pregnancy is uncommon among dialysis patients, with reported conception rates ranging from just 0.9% to 7% [2].

Fertility rates among women aged 18 to 49 with end-stage renal disease (ESRD) are approximately ten times lower than those observed in healthy women of the same age group [3]. Moreover, the presence of a chronic illness such as ESRD further exacerbates fertility challenges in affected women. Amenorrhea or irregular, typically anovulatory, menstrual cycles are frequently observed in this population [4–6].

Previous studies have demonstrated that kidney transplantation significantly restores reproductive function in patients with ESRD, with fertility rates improving by nearly fourfold compared to those undergoing dialysis [7–11]. Following kidney transplantation, fertility typically begins to normalize within a few months, and safe conception is generally considered feasible approximately one year post-transplant [8, 12]. Additionally, fertility may begin to improve as early as three weeks after transplantation, with the restoration of ovulatory cycles contributing to enhanced reproductive potential [13–15].

The first documented pregnancy in a transplant recipient occurred in 1957, when a woman who received a kidney transplant from her identical twin sister successfully delivered a healthy male infant [16].

Despite the encouraging improvements in fertility following kidney transplantation, recipients remain at elevated risk for adverse pregnancy outcomes. These include preterm delivery, low birth weight, and infants classified as small for gestational age, alongside maternal complications such as hypertension and preeclampsia [17, 18].

Therefore, it is essential that transplant recipients receive comprehensive counseling on factors associated with successful pregnancy outcomes. Key considerations include waiting at least one year post-transplant before attempting conception, demonstrating no evidence of graft rejection in the preceding year, maintaining stable graft function, being free from acute infections, adhering to stable maintenance doses of immunosuppressive therapy, and achieving well-controlled blood pressure [19, 20].

This study aims to evaluate maternal and fetal outcomes in pregnancies following kidney transplantation at King Faisal Specialist Hospital & Research Centre (KFSH&RC) between January 2012 and December 2021. By analyzing these outcomes, we seek to generate meaningful insights that will enhance patient counseling and inform evidence-based management strategies tailored to this unique patient population.

## Methods

### Study aim, design, and setting

This retrospective, single-center observational study was conducted at King Faisal Specialist Hospital and Research Centre (KFSH&RC), a tertiary care institution located in Riyadh, Saudi Arabia. The study was approved by the Institutional Review Board (IRB) at KFSH&RC, and all research procedures were carried out in accordance with relevant ethical standards and regulatory guidelines.

Electronic medical records of all pregnancies, along with up to two years of post-pregnancy follow-up, were reviewed for women who underwent kidney transplantation at King Faisal Specialist Hospital & Research Centre (KFSH&RC) between January 2012 and January 2021.

Patients who underwent kidney transplantation at external institutions, did not receive follow-up care at our center, or had a history of multi-organ transplantation were excluded from the study. Each pregnancy occurring post-transplant was treated as an independent observation, as maternal and neonatal outcomes can vary between pregnancies in the same individual.

### Patient demographics and clinical characteristics

Age, body mass index, comorbidities, type of donation (living or deceased), induction therapy details, and prior pregnancy history.

- Laboratory Data: Pre-pregnancy, pre-labor, and post-labor laboratory values, including creatinine, estimated glomerular filtration rate (eGFR) which is calculated using the CKD-EPI (Chronic Kidney Disease Epidemiology Collaboration) creatinine equation, complete blood counts, liver function tests, and tacrolimus levels.
- Pregnancy Details: Medications during pregnancy, pregnancy outcomes, complications, gestational age, and mode of delivery.
- Fetal Outcomes: Neonatal demographics, intensive care unit (ICU) admissions, neonatal weight, and any reported fetal complications.

Data access was restricted to the study authors, and no personally identifiable information was collected. Descriptive statistical analysis was performed using SPSS (IBM Corp. Released 2017. IBM SPSS Statistics for Windows). Categorical variables were summarized as frequencies and percentages, while continuous variables were reported using measures of central tendency, including means and medians.

### Multidisciplinary protocol for pregnancy in renal transplant recipients

At our hospital, the management of pregnancy in renal transplant recipients follows a structured, multidisciplinary protocol involving transplant physicians, obstetricians, maternal-fetal medicine (MFM) specialists, nephrologists, pharmacists, and neonatologists. Preconception counseling is initiated 6–12 months before planned pregnancy. We ensure candidates have stable graft function (eGFR ≥ 45 mL/min, stable creatinine), proteinuria < 500 mg/day, controlled blood pressure (< 130/80 mmHg on pregnancy-safe medications), and no acute rejection within the past six months. Teratogenic medications, including mycophenolate mofetil (MMF), are discontinued at least six weeks before conception and replaced with azathioprine. Ideally MMF is discontinued 2–3 months ahead of pregnancy. Tacrolimus is continued with a target trough of 5–8 ng/mL, and low-dose prednisolone (5–10 mg/day) is maintained. Tacrolimus levels are monitored every 2–4 weeks due to increased clearance, and serum creatinine, proteinuria, and blood pressure are checked monthly. Delivery planning is individualized. We aim for ≥ 37 weeks if both maternal and fetal conditions remain stable, though earlier delivery may be required for preeclampsia, worsening renal function, or fetal growth restriction. Vaginal delivery is preferred unless obstetric or graft-related indications necessitate cesarean section. Immunosuppressive medications are continued during labor, and stress-dose steroids are administered to patients on chronic prednisolone (> 5 mg/day).

In the postpartum period, our protocol involves continuing the immunosuppressive regimen, adjusting tacrolimus doses as pharmacokinetics normalize, and monitoring graft function closely. Breastfeeding is supported with tacrolimus, azathioprine, and prednisolone, but remains contraindicated with MMF. We also provide contraception counseling, favoring long-acting reversible methods, and comprehensive patient education on medication adherence, early warning signs of preeclampsia or rejection, and safe breastfeeding practices.

## Results

### Study population and baseline characteristics

This study evaluated a total of 37 pregnancies in 35 kidney transplant recipients. Each pregnancy post-transplant was treated as a separate observation in the analysis to ensure that every pregnancy outcome was evaluated independently. The mean maternal age was 29.6 ± 5.4 years. Most transplants were from living donors (88%), with (87%) involving related donors. Time between transplant and conception was 4.15 ± 2.37 years with no significant association with adverse fetal or maternal outcomes (p-value > 0.05). Only 6 out of 35 patients (17%) required dialysis prior to transplantation. Glomerulonephritis, including IgA nephropathy, focal segmental glomerulosclerosis (FSGS), membranous nephropathy, and lupus nephritis, was the most common etiology of ESRD, accounting for 17 cases (49%), followed by hypertension in 7 cases (20%), polycystic kidney disease in 6 cases (17%), obstructive uropathy in 3 cases (9%), and unknown cause in two cases (5%).

Comorbidities were observed in a number of patients, with hyperparathyroidism being the most prevalent (57%), followed by antiphospholipid syndrome (14%), hypertension (9%), and isolated cases of cauda equina syndrome, diabetes mellitus, spina bifida, and hyperthyroidism, each accounting for 5% of the cohort.

In terms of obstetric history, 24 (69%) of patients had previously been pregnant, while 11 (31%) reported a history of miscarriage, and 4 (12%) had undergone an abortion. In our study, we distinguished between spontaneous pregnancy loss (miscarriage) and induced/therapeutic abortion, as they represent clinically different scenarios with distinct implications for counseling and outcomes A detailed overview of patient characteristics is provided in Table 1.

**Table 1:** Characteristics of the study population. Data are presented as no. (%)

Patients Characteristics	No. 35 (%)
Age (Mean ± SD)	29.6 ± 5.4 years old
Time between transplant and conception (Mean ± SD)	4.15 ± 2.37 years
Living donor/Deceased donor	31 (88)/4 (12)
Related/non-related	27 (87)/4 (13)
Pre-transplant dialysis	6 (17)
Comorbidities	
Hyperparathyroidism	12
Antiphospholipid syndrome	3
Hypertension	2
Cauda Equina	1
Diabetes mellitus	1
Spina bifida	1
Hyperthyroidism	1
Previous Pregnancy	24 (69)
History of miscarriage/abortion	11 (31)/4 (12)

### Immunosuppressive therapy pre-pregnancy, during pregnancy, and postpartum

All patients were maintained on MMF prior to pregnancy, with 34 (97%) transitioning to azathioprine before conception in accordance with the established protocol. Of these, 29 (83%) continued azathioprine throughout the breastfeeding period, after which 100% of them were transitioned back to MMF.

### Laboratory parameters

Laboratory findings across various stages are presented in Table 2.

**Table 2:** Laboratory results. Data are presented as no. (%). ALT = Alanine Transaminase; AST,= Aspartate Aminotransferase; eGFR = Estimated Glomerular Filtration Rate

Labs	Within Normal Limits No. (%)	Abnormal levels No. (%)
Pre-Pregnancy Labs		
Hemoglobin	26 (74)	9 (26)
White blood cells	34 (97)	1 (3)
Platelets	35 (100)	-
Creatinine > 90 (µmol/L)	27 (77)	8 (23)
eGFR	29 (83)	6 (17)
Proteinuria	26 (74)	9 (26)
Bilirubin	35 (100)	-
Albumin	35 (100)	-
AST	35 (100)	-
ALT	35 (100)	-
Tacrolimus	7.4 ± 2.6	
Pre-Labor Labs		
Hemoglobin	12 (34)	23 (66)
White blood cells	30 (85)	5 (15)
Platelets	28 (79)	7 (21)
Creatinine > 90	20 (57)	15 (43)
eGFR	21 (60)	14 (40)
Proteinuria	22 (63)	13 (37)
Bilirubin	35 (100)	-
Albumin	35 (100)	-
AST	35 (100)	-
ALT	35 (100)	-
Tacrolimus	6 ± 3.6	
Post-Labor Labs		
Hemoglobin	18 (52)	17 (48)
White blood cells	29 (83)	6 (17)
Platelets	30 (85)	5 (15)
Creatinine	21 (60)	14 (40)
eGFR	22 (63)	13 (37)
Proteinuria	25 (71)	10 (29)
Bilirubin	35 (100)	-
Albumin	35 (100)	-
AST	35 (100)	-
ALT	35 (100)	-
Tacrolimus	6.84 ± 2.2	
Creatinine 6 months post-pregnancy	29 (83)	6 (17)
eGFR 6 months post-pregnancy	29 (83)	6 (17)
Creatinine 1-year post-pregnancy	26 (74)	9 (26)
eGFR 1-year post-pregnancy	26 (74)	9 (26)
Creatinine 2-year post-pregnancy	28 (79)	7 (21)
eGFR 2-year post-pregnancy	28 (79)	7 (21)

During the pre-pregnancy phase, most patients exhibited laboratory values within normal ranges. However, 9 (26%) presented with anemia,8 (23%) had elevated serum creatinine levels (> 90 µmol/L), and 6 (17%) showed a reduced estimated glomerular filtration rate (eGFR) of < 60 mL/min/1.73 m². The mean creatinine in pre-pregnancy was 81.7 ± 18.8 µmol/L with a mean eGFR of 70.4 ± 13.4 mL/min/1.73 m².

By the pre-labor period, a noticeable decline in laboratory parameters was observed. Anemia affected 66% of patients, platelet abnormalities were seen in 21%, and 15% had elevated white blood cell counts. Renal function also deteriorated, with 43% exhibiting elevated creatinine levels and 40% demonstrating a decrease in eGFR below 60 mL/min/1.73 m², indicating a decline in graft function. Mean tacrolimus levels decreased from 7.4 ± 2.6 ng/mL pre-pregnancy to 6.0 ± 3.6 ng/mL prior to labor. The mean creatinine in pre-pregnancy was 94.7 ± 29.7 µmol/L with a mean eGFR of 63.5 ± 17.6 mL/min/1.73 m².

In the post-labor period, 48% of patients remained anemic, 40% continued to show elevated creatinine levels, and 37% had persistently reduced eGFR. The proportion of patients with elevated white blood cell counts rose to 17%, while the proportion of patients with abnormal platelet levels decreased to 15%. The mean creatinine in pre-pregnancy was 88.9 ± 26.9 µmol/L with a mean eGFR of 64.8 ± 15.1 mL/min/1.73 m².

### Long term renal function

**Fig. 1:**
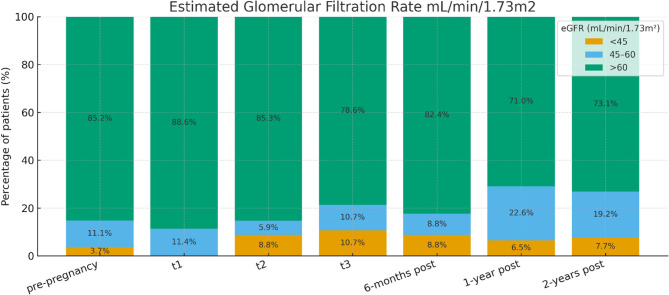
Estimated glomerular filtration rate before, during, and after pregnancy. T1: term 1 of pregnancy; T2: term 2 of pregnancy; T3: term 3 of pregnancy

At six months postpartum, renal function had notably improved in the majority of patients, with 29 (83%) maintaining normal creatinine and eGFR levels. However, by one year postpartum, a modest decline was observed, with 26% of patients exhibiting reduced eGFR and elevated creatinine levels. At the two-year follow-up, 21% continued to demonstrate impaired renal function. Trends in eGFR levels before, during, and after pregnancy are illustrated in Fig. 1.

### Pregnancy and neonatal outcomes

As detailed in Table 3, the mean gestational age at delivery was 35.27 ± 5.74 weeks. Over half of the pregnancies (57%) reached full term, while 29% resulted in moderate to late preterm deliveries (32 to < 37 weeks). Additionally, 8% were classified as extremely preterm (< 28 weeks), and 6% as very preterm (28 to < 32 weeks). Two pregnancies ended in miscarriage. Among the cohort, only one patient experienced a multiple gestation, while the remaining 34 had singleton pregnancies.

**Table 3:** Pregnancy and neonatal outcomes. Data are presented as no. (%)

Pregnancy variables	No. 35 (%)
Gestational age (mean ± SD)	35.27 ± 5.74 weeks
Gestational age subcategory	
Extremely preterm (< 28 weeks)	3 (8)
Very preterm (28 to < 32 weeks)	2 (6)
Moderate to late preterm (32 to < 37 weeks)	10 (29)
Term (≥ 37 weeks)	20 (57)
Singleton pregnancy	34 (97)
Multiple pregnancy	1 (3)
Immunosuppressive therapy	
Tacrolimus-Azathioprine-Prednisolone	32 (91)
Azathioprine-Prednisolone	2 (6)
Tacrolimus-Prednisolone	1 (3)
Aspirin use	17 (49)
Miscarriage	2 (6)
Pregnancy complications	
Preeclampsia	1 (3)
Gestational hypertension	1 (3)
Gestational diabetes mellitus	1 (3)
Intrauterine growth restriction	1 (3)
Retained products of conception	1 (3)
Placenta previa	1 (3)
Mode of Delivery	
Cesarean section/Spontaneous vaginal delivery	26 (74)/9 (26)
Indication for caesarean section	
Fetal distress	8
Previous caesarean section	4
Failed induction	3
Elective	3
Preeclampsia	3
Premature rupture of the membranes	1
Placenta previa	1
Placenta abruptio	1
Herpes simplex virus infection	1
Cephalic/Breech presentation	33 (94)/2(6)
Blood loss < 500/Blood loss ≥ 500	19 (54)/16 (46)
Hospital length of stay (Days)	2.79 ± 1.82 days
Neonatal Outcomes	
Required ICU	9 (26)
Congenital abnormalities	1 (3)
Very low birth weight (< 1500 g)	2 (6)
Moderately low birth weight (1500 to 2500 g)	11 (31)
Normal birth weight (> 2500 g)	22 (63)

### Immunosuppressive regimens

The majority of patients (91%) were managed with a Tacrolimus–Azathioprine–Prednisolone immunosuppressive regimen. Alternative regimens included Tacrolimus–Prednisolone (3%), Azathioprine–Prednisolone (3%), and Hydroxychloroquine–Prednisolone (3%). Additionally, aspirin was administered in 49% of pregnancies.

### Pregnancy complications

Pregnancy complications were observed in 14% of cases, including preeclampsia (3%), gestational hypertension (3%), gestational diabetes (3%), intrauterine growth restriction (3%), and placenta previa (3%). The lower incidence of hypertension and preeclampsia in our cohort likely reflects strict preconception selection, with pregnancy deferred until graft function, blood pressure, and proteinuria were well controlled.

### Delivery outcomes

Caesarean section (C-section) was performed in 74% of patients, with fetal distress being the most common indication (31%). Other indications included a history of previous C-Sect. (15%), failed induction (12%), elective C-section, and preeclampsia (12%). Less frequent indications comprised premature rupture of membranes (4%), placenta previa (4%), placental abruption (4%), and herpes simplex virus infection (4%). Notably, 46% of deliveries were associated with blood loss of ≥ 500 mL. The mean length of hospital stay was 2.79 ± 1.82 days.

### Neonatal outcomes

A total of 9 (26%) of neonates required admission to the intensive care unit (ICU), and 1 (3%) was found to have congenital abnormalities. In terms of birth weight, 22 (63%) were born with a normal weight (> 2500 g), while 11 (31%) had moderately low birth weight (1500–2500 g), and 2 (6%) were classified as having very low birth weight (< 1500 g).

### Subgroup analysis

Subgroup analysis examined associations between donor type, maternal comorbidities, and pregnancy outcomes in 35 kidney transplant recipients. Fisher’s exact test was used for all comparisons due to small sample sizes. Deceased donor recipients had significantly worse outcomes than living related donors, with 100% pregnancy complications (4/4) versus 22.2% (6/27), respectively (Fisher’s exact, *p* < 0.05). No significant differences between donors when it came to fetal outcomes (Fisher’s exact, *p* > 0.05). Comorbidities were not significantly associated with fetal or maternal outcomes (Fisher’s exact, *p* > 0.05).

## Discussion

Although numerous studies have investigated maternal and fetal outcomes in pregnant women following renal transplantation, the existing literature remains heterogeneous and is often constrained by small sample sizes, outdated data, and geographic biases in publication [21]. Few studies in the literature have comprehensively evaluated pregnancy outcomes while simultaneously assessing the long-term impact on graft function, highlighting a significant gap in current research [22–26] with one study assessing only in vitro fertilization outcomes [24]. Over the past two decades, research on pregnancy outcomes in kidney transplant recipients within the Middle East has been notably scarce [25–27]. In Saudi Arabia, only a single study has been conducted, featuring a limited cohort of 12 patients and published over 15 years ago, underscoring the need for more contemporary and region-specific data [28]. The maternal age in our study is consistent with findings from previous literature [29–31], however, some studies have reported a higher mean maternal age [32, 33]. Limited studies have examined comorbidities within this specific patient population. While hyperparathyroidism emerged as the most prevalent comorbidity in our cohort, other studies have more commonly reported diabetes mellitus and hypertension as the predominant coexisting conditions [31, 34]. Renal function exhibited a progressive decline throughout pregnancy, most notably during the pre-labor period, as indicated by elevated creatinine levels (>90 µmol/L in 43% of patients) and reduced eGFR (< 60 mL/min/1.73 m² in 40%). Unlike previous studies that primarily reported mean creatinine [35–38] and mean eGFR [39, 40] our study adopts a more detailed approach by categorizing patients based on normal versus abnormal renal function. This stratification provides a clearer and more clinically meaningful understanding of the proportion of pregnant women experiencing renal deterioration, offering a nuanced perspective on kidney function trends during pregnancy. Tacrolimus levels declined from a pre-pregnancy mean of 7.4 ± 2.6 ng/mL to 6.0 ± 3.6 ng/mL prior to labor, likely to reflect the increased metabolic rate and expanded volume of distribution characteristic of pregnancy. This finding aligns with the limited number of studies in the literature that have examined tacrolimus pharmacokinetics during pregnancy [39, 41].

In our study, the most frequently encountered issues—such as pregnancy-induced hypertension, preeclampsia, preterm birth, low birth weight, and a high rate of cesarean deliveries were consistent with findings from previous research, however other studies reported higher rates of preeclampsia and gestational hypertension [42, 43]. Notably, however, our cohort demonstrated a significantly higher live birth rate, distinguishing it from earlier reports and suggesting more favorable outcomes in this population [43]. To the best of our knowledge, this is the only study to date that reports on fetal presentation in kidney transplant recipients, identifying breech presentation in just 6% of cases. Notably, none of the patients in our cohort developed acute or chronic graft rejection, even within the first two years postpartum. Moreover, creatinine and eGFR levels demonstrated a clear trend toward normalization after pregnancy, returning close to their pre-pregnancy baselines, findings that align with recent evidence suggesting minimal long-term impact on graft survival [44]. Nonetheless, the long-term impact of pregnancy on graft function remains insufficiently explored, with existing studies characterized by methodological variability and limited follow-up. Existing studies are few and vary significantly in design, making direct comparisons challenging. Notably, those studies only provide the mean eGFR and creatinine levels with no comprehensive analysis of the number of patients with persistent renal dysfunction during follow-up years [35, 45]. While that might be the case, the studies did end up offering reassuring results similar to our findings on the long-term effect of pregnancy on the graft function.

## Limitations

This study has several limitations that should be taken into account when interpreting the results. Firstly, its retrospective design inherently restricts control over confounding variables and introduces the potential for selection bias. Retrospective nature also limited the ability to have laboratory confirmation for certain variables such as hyperparathyroidism as a comorbidity. Secondly, as a single-center study conducted at a tertiary care institution, the findings may not be fully generalizable to broader populations, particularly those in settings with differing healthcare systems and management practices. While the sample size is comparable to other studies in this field, it remains relatively small, limiting statistical power and potentially underrepresenting rare complications or long-term outcomes.

Additionally, the absence of a control group consisting of non-pregnant kidney transplant recipients restricts the ability to isolate the specific impact of pregnancy on graft function. Although renal function was monitored postpartum, the follow-up period may not have been long enough to fully capture the long-term effects of pregnancy on graft survival and maternal health.

## Conclusion

This study offers important insights into the maternal and fetal outcomes of pregnancies among kidney transplant recipients within a Middle Eastern cohort. The results reaffirm that, although pregnancy in this population remains high-risk, the majority of patients experience favorable outcomes, highlighted by a notably high live birth rate. Renal function showed a temporary decline during pregnancy; however, most patients demonstrated a return to near pre-pregnancy levels postpartum. Significantly, no instances of acute or chronic graft rejection were observed, underscoring the safety of pregnancy in carefully selected transplant recipients with stable graft function. Nonetheless, to better understand the long-term implications of pregnancy on graft durability and maternal health, larger, multicenter studies with extended follow-up and standardized methodologies are warranted.

## Data Availability

The data that support the findings of this study are not openly available due to reasons of sensitivity and are available from the corresponding author upon reasonable request.

## Abbreviations

ESRD: End stage renal disease
eGFR: Estimated glomerular filtration rate
ICU: Intensive care unit
AST: Aspartate Aminotransferase
ALT: Alanine aminotransferase
